# Thermal performance of scleractinian corals along a latitudinal gradient on the Great Barrier Reef

**DOI:** 10.1098/rstb.2018.0546

**Published:** 2019-06-17

**Authors:** S. Jurriaans, M. O. Hoogenboom

**Affiliations:** 1 Marine Biology and Aquaculture, College of Science and Engineering, James Cook University, Townsville, Queensland, Australia; 2 ARC Centre of Excellence for Coral Reef Studies, James Cook University, Townsville, Queensland, Australia

**Keywords:** thermal heterogeneity, thermal performance curve, respirometry, acclimatization, plasticity, reaction norm

## Abstract

Species have evolved different mechanisms to cope with spatial and temporal temperature variability. Species with broad geographical distributions may be thermal generalists that perform well across a broad range of temperatures, or they might contain subpopulations of locally adapted thermal specialists. We quantified the variation in thermal performance of two coral species, *Porites cylindrica* and *Acropora* spp., along a latitudinal gradient over which temperature varies by approximately 6°C. Photosynthesis rates, respiration rates, maximum quantum yield and maximum electron transport rates were measured on coral fragments exposed to an acute temperature increase and decrease up to 5°C above and below the local average temperature. Results showed geographical variation in the performance curves of both species at holobiont and symbiont level, but this did not lead to an alignment of the optimal temperature for performance with the average temperature of the local environment, suggesting suboptimal coral performance of these coral populations in summer. Furthermore, symbiont thermal performance generally had an optimum closer to the average environmental temperature than holobiont performance, suggesting that symbionts have a higher capacity for acclimatization than the coral host, and can aid the coral host when temperatures are unfavourable.

This article is part of the theme issue ‘Physiological diversity, biodiversity patterns and global climate change: testing key hypotheses involving temperature and oxygen’.

## 1. Introduction

Many species have wide geographical distributions that cover broad latitudinal gradients and a correspondingly broad range of environmental conditions. For instance, populations that reside at higher latitudes are exposed to colder environments than populations that occur around the equator [1], and the thermal environment is generally more variable at higher latitudes compared with at the equator [2]. To cope with thermal heterogeneity along latitudinal gradients, species have evolved different thermal responses associated with a wide range of physiological, morphological and behavioural traits [3]. Consequently, two species may tolerate a similar range of temperatures, and occupy the same geographical range, using very different mechanisms to cope with temperature variation. As climate change scenarios predict increased fluctuations in temperature and thermal extremes [4], there has been an increased focus on the impacts of climatic variability on the physiology and ecology of individuals, populations and communities [5] and generally suggest that plasticity increases in more variable environments [6], although many studies failed to incorporate extreme events [7]. The relationship between temperature and a trait can be fixed or (more or less) plastic along a temperature gradient [3,8]. Plasticity of this relationship may lead to thermal acclimatization, defined as the adjustment of a physiological trait in response to changes in the environmental temperature that alters the performance to enhance fitness (not to be confused with ‘acclimation’ which refers to physiological responses to changes in an environmental variable in the laboratory [9]). As such, thermal acclimatization can be reversible and occur constantly throughout an organism's life. However, if physiological adjustments occur during early life, the changes can become fixed during the life of the organism (known as ‘developmental plasticity’ [10]). When the relationship between temperature and performance is fixed, the species requires a broad thermal tolerance according to the entire temperature gradient that is encountered throughout its geographical distribution. This thermal generalist strategy is likely to occur if gene flow among local populations prevents local adaptation [11], if temperature fluctuations are rapid and unpredictable making acclimatization ineffective [12], or if the costs of plasticity outweigh the benefits [13]. Alternatively, a species might select specific thermal microhabitats within its geographical range, by way of behaviour or through habitat selection at settlement, such that it experiences a homogeneous thermal environment and plasticity is not required. Such thermal specialist species can be expected to have higher maximal performance than thermal generalists [14]. Lastly, a species could perceive the thermal environment as heterogeneous *among* populations, but homogeneous *within* populations [12]. Such species can maximize performance within each population through thermal acclimatization, and/or local adaptation in cases where populations are isolated, and can be referred to as ‘plastic’ thermal specialists. Consequently, a plastic thermal specialist species can survive under a similar range of temperatures to that of a thermal generalist species, but uses a very different strategy to do so.

Thermal performance curves (TPCs) are widely used to quantify the thermal sensitivity of species (see review by Angilletta [3]). TPCs show the instantaneous performance of an organism in response to short-term (acute) environmental fluctuations along a temperature gradient [15]. Typically, this produces a curve from which three important parameters can be derived [16]: the maximal performance (Pf_max_), the temperature for optimal performance (*T*_opt_) and the temperature range over which the performance is positive, known as the thermal breadth (*T*_br_). Through thermal acclimatization, the shape and position of the curve can change in response to changes in the thermal environment [17]. Each shift represents a trade-off between the cost of acclimatization and the benefit gained from enhancing performance in the changed environment [18]. For instance, increasing *T*_opt_ will enhance performance in warm environments, but can be costly if the environmental temperature decreases unpredictably. Thus, to maximize performance, it is important to adopt a thermal strategy that corresponds to the present and future thermal environment. Because nearly all environments vary both within and among populations, particularly for long-lived species with wide geographical distributions, optimality models predict that shifting *T*_opt_ through developmental plasticity is only beneficial if the thermal heterogeneity among sites is greater than within sites [19], and environmental cues are accurate [20]. Additionally, increasing *T*_br_ is only beneficial if the temperature does indeed fluctuate during the organism's lifetime, because increased thermal breadth comes at the cost of reduced Pf_max_ [14]. In summary, the thermal generalist strategy enables species to have positive performance across a wide temperature range, but allows for misinterpretation of environmental cues. By contrast, developmental plasticity allows a plastic thermal specialist to maximize performance within a narrow temperature range but comes at the cost of poor performance when environmental cues are not accurate. Lastly, TPCs vary between traits owing to different proximate mechanisms that underlie the phenotypic expression [18]. Therefore, the performance of multiple traits at various levels of physiological organization should be measured when comparing TPCs of populations along a latitudinal cline.

Corals reefs are among the most productive and biologically diverse ecosystems on Earth [21]. The Great Barrier Reef (GBR) off the coast in northeastern Australia is the world's largest coral reef ecosystem containing approximately 3000 individual reefs extending over 14° of latitude. Accordingly, there is a thermal gradient along the GBR with a cooler and more variable thermal environment in the southern GBR and a warmer and more stable thermal environment towards the northern GBR [22], yet many hard coral species have distribution ranges throughout the entire GBR [23]. Consequently, the thermal environment these species experience varies significantly across space and through time, while their thermal strategy is largely unknown. This is partly because studies of coral thermal biology focused on identifying the upper thermal thresholds for coral bleaching (i.e. the breakdown of the symbiosis between corals and their photosynthetic algae), e.g. [24–26]. Additionally, studies that investigated coral performance over a temperature gradient are ambiguous about the species-specific and environmental controls on coral thermal tolerance. For instance, *T*_opt_ for growth of the tropical species *Pocillopora damicornis* varied between populations with different thermal environments [27], suggesting a plastic thermal specialist strategy, whereas Castillo & Helmuth [28] showed no difference in *T*_opt_ for net productivity of *Montastraea annularis* among populations. A more recent study [29] showed no variation in the *T*_opt_ for multiple coral host and symbiont-related performance traits for Mediterranean corals from populations with different thermal environments, suggesting a thermal generalist strategy. Studies comparing the thermal performance of multiple coral species, and across multiple physiological traits, are required to assess whether and how thermal tolerance strategies differ among species.

The overarching aim of this study was to determine whether and how the coral thermal physiology varies between species and among populations distributed along a latitudinal gradient in the GBR over which temperature varies by approximately 6°C, and thereby assess their thermal performance. We investigated the shape and position of the TPC of two coral species from three populations with different thermal environments. This allowed us to answer whether these populations were acclimated to their specific thermal environment, suggesting a plastic thermal specialist strategy, or if they shared a common TPC, suggesting a thermal generalist strategy. Additionally, we investigated the variation in the thermal strategy between species with a similar thermal range across their geographical distribution. Assuming that there was limited gene flow between reefs [30], the TPCs should vary predictably along the latitudinal gradient. We hypothesized that corals from the southern reef have their *T*_opt_ at a lower temperature than corals from the central or northern reefs, and that the *T*_br_ increases with increasing thermal heterogeneity. Knowledge of the plasticity of the thermal performance of coral species and their thermal tolerance strategies will provide insight into how global warming might shape coral reefs, as a plastic thermal specialist strategy may result in higher fitness under global warming than a thermal generalist strategy.

## 2. Material and methods

### (a) Experimental design

**Figure 1.**
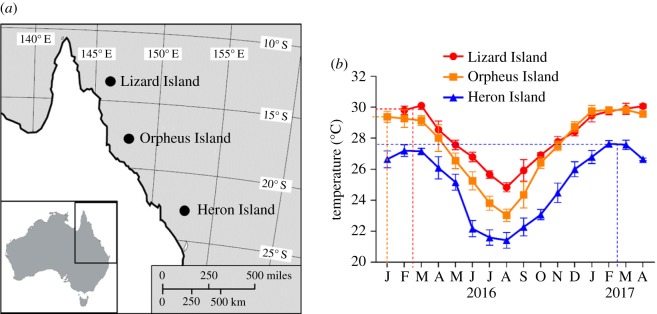
Coral collection and experimental study sites located along a latitudinal gradient in Great Barrier Reef (*a*) and monthly average seawater temperatures (*b*). Dashed lines indicate the average ambient temperatures at the start of the thermal experiment for each location. Data sourced from AIMS 2017 [22].

The study locations (figure 1*a*) occurred along a latitudinal gradient between Lizard Island (LI) situated in the northern GBR (14° 40′08″ S, 145° 27′34″ E), Orpheus Island (OI) in the central GBR (18° 37′06″ S, 146° 29′37″ E) and Heron Island (HI) in the southern GBR (23°26'18.71″ S, 151°54'30.23″ E). LI and HI are both further offshore (approx. 30 km and approx. 80 km, respectively) compared with OI (approx. 17 km), and the latter generally experiences higher turbidity. The temperature along this gradient varies by approximately 6°C during the summer months, ranging from approximately 24°C at HI to approximately 30°C at OI and LI. Seawater temperature data from December 2015 to March 2017 were recorded by *in situ* data loggers deployed by the Australian Institute of Marine Science (AIMS) [22] at LI, OI and HI at a depth of 10.1 m, 5.8 m and 5.4 m, respectively.

Fragments of *Acropora intermedia* (at HI and LI), *Acropora valenciennesi* (at OI) and *Porites cylindrica* (at LI, OI and HI) were collected. Two species of *Acropora* were sampled owing to their local abundances at the study locations. Both species have similar morphologies (arborescent branching), contain similar symbiont species *Cladocopium* C3 [31–33]) and are sensitive to high temperatures [34], whereas *P. cylindrica* contains *Cladocopium* C15 [31–33] and is more tolerant to high temperatures [24]. Between-genus differences in the shape and position of the TPCs were therefore expected. Twenty-five fragments of each species (approx. 8 cm tall, 5 per colony) were collected by hand using a bone cutter at depths between 4 and 6 m by SCUBA diving. Fragments were collected from the top of the mother colony to minimize variation in the light environment between colonies. The duration of the thermal experiments meant that data collection could not be collected at all locations in the same season in a single year, consequently corals were collected in November/December 2015 at OI (thermal experiment starting 25 January 2016), mid-February 2016 at LI (thermal experiment starting 2 March 2016) and mid-February 2017 at HI (thermal experiment starting 6 March 2017).

After collection, fragments were transported to the research station situated on the island, attached to nylon string, labelled to keep track of colony identity and randomly distributed among two adjacent large (50 l) shaded outdoor tanks with two fragments of the same colony per tank. Both tanks received a constant supply of seawater pumped from the adjacent reef flat at equal inflow rates (approx. 90 l h^−1^). Therefore, the corals experienced the same light environment and water chemistry between tanks. The average (and maximum) seawater temperature measured over two weeks prior to the start of the thermal experiment on the reef flat was 29.9 (30.7)°C at LI, 29.3 (30.2)°C at OI and 27.6 (28.8)°C at HI (figure 1*b*). Fragments were given at least two weeks to recover from collection and acclimate to the tank conditions before starting the measurements.

Care was taken to minimize variation in the experimental procedure at each research station and the following description of the thermal experiment applies to each location, unless specified. Corals were divided into two groups (two fragments of each colony per group); one group was exposed to progressively lower temperatures, while the other group was exposed to progressively higher temperatures. This design enabled calculation of two TPCs per colony over the entire temperature gradient. Coral performance (described below) was first measured at ambient temperature, after which one fragment of each colony was immediately frozen at −80°C (*n* = 5) for tissue analyses. After that, the water temperature in each tank was increased, or decreased, each day by 0.5°C using a chiller/heater unit (TK-2000, TECO, Italy) connected to a pump (Aquapro AP1050, Aquatec, Australia) that circulated the water at a rate of 500 l h^−1^. This continued for 10 days, resulting in a total temperature change of 5°C above and below ambient temperature. At every 1°C increment, several response variables were measured as an indicator of the acute coral performance (or instantaneous thermal sensitivity [4]) at that temperature. Qualitative observations were made about the coral colour (paleness) and tentacle expansion of the fragments in the holding tank twice per day (morning and evening). At the end of the thermal experiment, fragments were frozen at −80°C and transported to laboratory facilities at James Cook University for tissue analyses.

### (b) Coral performance

Different response variables were measured to differentiate between the thermal responses of the holobiont versus the photosynthetic symbionts specifically. Net photosynthesis and respiration rates, measured using oxygen respirometry, are mostly dominated by the coral host physiology because the biomass of the coral tissue is much larger than the biomass of the symbionts [35]. Maximum quantum yield and electron transport rate were measured using fluorometry, as a proxy for the symbiont response, because this measuring technique quantifies the fluorescence signal from the photosynthetic pigments within the symbionts specifically.

### (c) Holobiont response variables

Net photosynthesis (Pn) and respiration (*R*) rates of the coral fragments were measured in transparent experimental cells (six cells, 550 ± 5 ml). Five cells contained filtered (15 µm) seawater and one coral fragment (suspended on a nylon string in the upright position similar to the holding tank), and a separate control cell contained only filtered seawater to account for background respiration of microorganisms in the seawater. The cells were placed on a submersible magnetic stirrer plate (MIXdrive 6, 2mag, Germany) in a water bath that controlled the water temperature inside the cells. A magnetic stirrer bar inside each cell ensured continuous mixing of the water to prevent diffusion limitation of gas exchange [36]. The temperature of the water bath was controlled by a chiller/heater unit (TK-2000, TECO). Care was taken to minimize air exposure and manual handling of coral fragments during transfer to the respirometry chambers. Fifteen minutes after placing the corals in their incubation chambers, the dissolved oxygen concentration inside each cell was measured at 1 min intervals for 1 h using optical dissolved oxygen sensors (LDO101, Hach, USA) connected to a meter device (HQ40D, Hach). Oxygen sensors were factory calibrated and there was no indication of drift over time. Pn rates were measured at a light intensity of 350 µmol photons m^−2^ s^−1^ provided by LED lights (R420r, 180 W, Maxspect Razor). At OI, two wide beam lamps (Oracle Sylvania, Australia) with 150 W metal halide bulbs were used. Irradiance was at 350 µmol photons m^−2^ s^−1^ measured with LI-193 Spherical Underwater Quantum Sensor (LI-COR, USA). This irradiance level is within the range of what corals naturally experience at HI, OI and LI (electronic supplementary material, table S1) and approximates saturating irradiance for various coral species across the GBR (e.g. [37–41]). After the photosynthesis measurement, the room was darkened and the corals were given 15 min to acclimate to darkness before measuring *R* rates during 1 h. Afterwards, corals were returned to their holding tanks. Pn and *R* rates were corrected for background oxygen consumption/production by subtracting the differential oxygen concentration of the empty control cell, and multiplying by the water volume of the cell. Data were normalized by coral skeletal surface area using the wax dipping method described by Veal *et al.* [42].

### (d) Symbiont response variables

After the dark respirometry, the maximum quantum yield (*F*_v_/*F*_m_) of photosystem (PS) II was measured on the dark-adapted fragments using a pulse-amplitude modulated fluorometer (DIVING-PAM, Walz, Germany). *F*_v_/*F*_m_ describes the maximum capacity of open PS II reaction centres (within the symbiont) to capture light energy for photosynthesis [43]. The quantification of *F*_v_/*F*_m_ over a temperature gradient provides an indication of the PS II activity, or ‘performance’, of the symbiont at each temperature increment. Minimum and maximum chlorophyll fluorescence (respectively, *F*_0_ and *F*_m_) were measured with a fibreoptic probe at a fixed distance (approx. 3 mm) from the coral surface. *F*_v_/*F*_m­_ was calculated as [*F*_m­_ – *F*_0_]/*F*_m_ [44]. On each coral fragment, five measurements, evenly distributed over the coral surface, were made from which an average *F*_v_/*F*_m_ was calculated. Corals were assumed to be dark-adapted after 40 min in darkness [45].

After the light respirometry, rapid light curves (RLCs) were measured on the light-adapted fragments using the DIVING-PAM. RLCs provide information on the saturation characteristics of the electron transport and the photosynthetic performance of the symbiont [46]. Here, RLCs were used to assess the photosynthetic capacity of PS II at different temperatures as a function of instantaneous irradiance after illumination for a fixed time period. RLCs were measured using an internal program of the DIVING-PAM that provided a sequence of nine light steps with light intensities increasing from 5 to 1800 µmol photons m^−2^ s^−1^. Each illumination period lasted 10 s and finished with a saturating pulse that measured the effective quantum yield (Δ*F*/*F*_m_′) of the light-adapted sample. The relative electron transport rate (rETR) was then calculated as:

2.1 $$\mathrm{rETR}=\frac{\Delta F}{{F_{m}}^{\prime }}\times \mathrm{PAR}\times 0.5,$$

where PAR is the photosynthetically active radiation and 0.5 corrects for two photons of light required for the transport of one electron. RLCs were created by plotting rETR against instant irradiance, from which the maximum rETR (rETR_m_) was taken.

### (e) Chlorophyll concentration

Chlorophyll concentrations were measured in fragments sampled at the start of the experiment (ambient group, *n* = 5) and the end of the experiment (heated and chilled group, *n* = 10 per group). Coral tissue was removed from the skeleton using an airbrush and 15 ml filtered (15 µm) seawater. The tissue slurry was homogenized using a homogenizer (T25 Ultra-Turrax, IKA, Germany) and centrifuged for 10 min at 5000*g* (Rotina 380R, Hettich Lab Technology, Germany). The supernatant was discarded and 5 ml of 90% acetone was added to the pellet and left in darkness overnight at 4°C. Absorbance was measured at 630, 663 and 750 nm using a spectrophotometer (Spectramax M2 Reader, Molecular Devices, USA). Chlorophyll *a* and *c*2 concentrations were calculated using equations of Jeffrey & Humphrey [47] and normalized by skeletal surface area.

### (f) Data analyses

Data were analysed using the statistical software R v. 3.0.3 (The R Foundation for Statistical Computing).

To assess whether the temperature response of *P. cylindrica* and *Acropora* spp. varied between locations and species, nonlinear least-squares regression models were fitted to the data for each response variable (Pn, *R*, *F*_v_/*F*_m_ and rETR_m­_). A symmetrical Gaussian function [29] was chosen over an asymmetrical function as this provided a better fit with fewer parameters [48]:

2.2 $$P=Pf_{\max}\exp\;\left[-0.5{\left(\frac{\mathrm{abs}\;(T\text{–}T_{\mathrm{opt}})}{T_{\mathrm{br}}}\right)}^{2}\right],$$

where *P* is the temperature-dependent physiological response, Pf_max_ is the maximum value of that response, *T*_opt_ is the temperature at which the response value is optimal (i.e. the mean value) and *T*_br_ provides a measure of the breadth of the response curve (i.e. the standard deviation).

For each response variable, the function was first fitted to the data regardless of location and species, then fitted to the data separated by either species or location, then to the data separated by both species and location, and finally to the data separately for each coral colony of each species and at each location. The Akaike information criterion (AIC) was used to assess whether the shape of the TPC differed significantly between species and among locations. We summed the AIC values over the multiple fits of the equation to different divisions of the data, and chose the division of the data with the lowest AIC value as the model that was most strongly supported by the data.

As the overall aim of this study was to determine whether coral populations are acclimatized and/or adapted to the thermal regime of their local environment, we focused primarily on the average responses of the species at each site. Therefore, the population response was calculated for each parameter of the TPC (*P*_max_, *T*_opt_ and *T*_br_) by averaging the colony responses at every location (per species). A one-way analyses of variance (ANOVA) was used to detect differences in the parameter estimations between the populations. When there were significant differences, Tukey post hoc analyses were performed. *p*-values were considered significant when *p* < 0.05.

Chlorophyll data were tested for assumptions of normality using the Shapiro–Wilk test and Levene's test for homogeneity of variance and log or square root transformed when the assumption of homogeneity was violated. To detect differences in the mean chlorophyll concentrations within species across location and treatment, data were analysed using mixed-effects ANOVAs with treatment (heated and chilled) and location as fixed effects and colony as random effect. Chlorophyll concentrations of the fragments collected at the start of the experiment at ambient temperature were analysed separately, using a two-way ANOVA with species and location as main effect, to detect differences in the chlorophyll concentration between locations and species.

## 3. Results

### (a) Thermal environment at study locations

The average (and maximum) seawater temperature was 29.4 (30.2)°C at OI in January 2016, 29.7 (30.7)°C at LI in February 2016 and 27.6 (29.1)°C at HI in February 2017. Temperature data were not available for LI during December 2015 and January 2016, but overall, seawater temperatures were distinctly lower at HI compared with OI and LI, with the latter two sites having similar summer temperatures (electronic supplementary material, table S2). However, in winter, OI experienced cooler temperatures than LI and therefore, the annual variability in temperature was larger at OI than at LI (minimum and maximum temperature in 2016/2017 at OI was 22.2°C to 31.0°C and at LI 24.2°C to 30.8°C). The annual temperature variability was even greater at HI, where temperature fluctuated from 18.1°C to 29.1°C in 2016/2017, which was 1.7 times larger than the fluctuation at LI and 1.3 times larger than that at OI.

### (b) Thermal performance

The corals showed high survival during the experiments, with 93% remaining alive at the end of the thermal experiment. At LI, all four fragments from one *Acropora* colony showed tissue necrosis after *T*_exp_ + 3°C and *T*_exp_ − 4°C. These fragments were excluded from the experiment because the cause of the tissue necrosis could not be reliably determined. Some paling of tissues was observed for *Acropora* fragments at both HI and LI, when the experimental temperature reached *T*_exp_ + 5°C and tentacle expansion was no longer observed at those temperatures.

**Figure 2.**
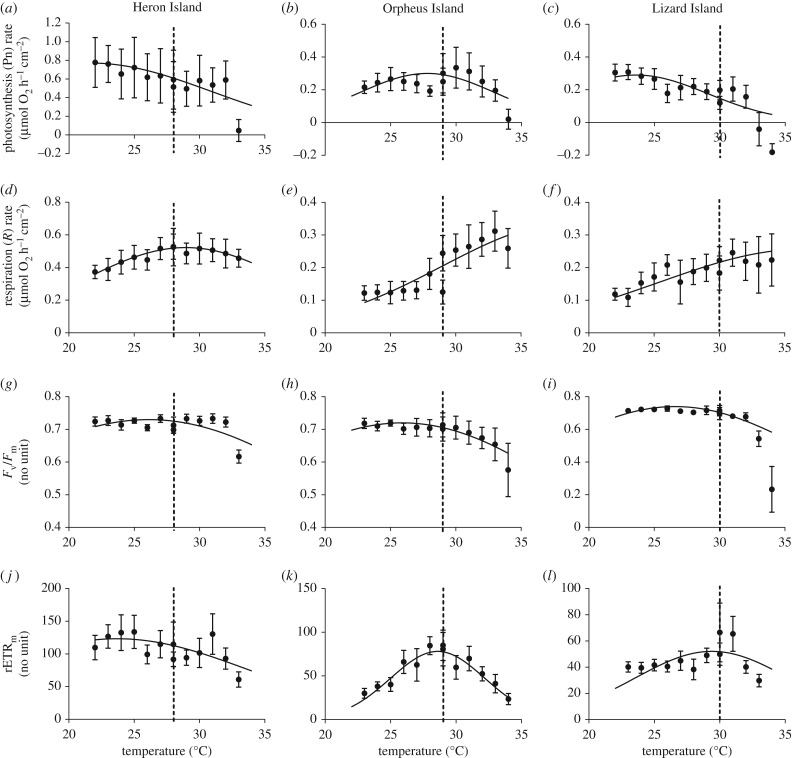
TPCs of *Acropora* spp. measured at HI, OI and LI. Datapoints are the mean values ± s.d. (*n* = 10). Curves were fitted with least square nonlinear regressions using equation (2.1).

**Figure 3.**
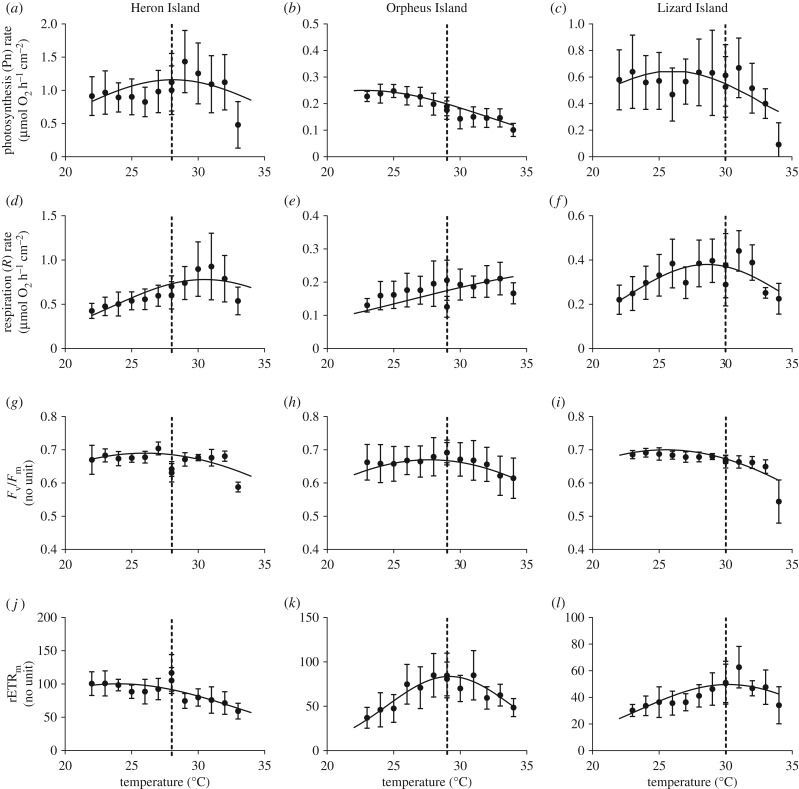
TPCs of *P. cylindrica* measured at HI, OI and LI. Datapoints are the mean values ± s.d. (*n* = 10). Curves were fitted with least square nonlinear regressions using equation (2.1).

The response variables generally showed nonlinear relationships with temperature for both *Acropora* (figure 2) and *Porites* (figure 3). Model selection based on AIC revealed that data divided by location, by species and by individual coral colony provided the best fit to the host and symbiont response variables (electronic supplementary material, table S3). Dividing the data by location and by species provided the next best fit to the data and the model selection technique did not support pooling data across locations or across species, which indicates that the thermal performance varied among locations and between species.

### (c) Holobiont response

The temperature at which Pn was maximum, *T*_opt_, was below the environmental temperature at all three locations for both species, except for the *Porites* population at HI where the optimal temperature was approximately the same as the environmental temperature (figure 3*a*). There was no clear trend of increasing *T*_opt_ corresponding to increasing environmental temperature for either species; for *Acropora*, the highest *T*_opt_ was observed at OI (27.8 ± 1.5°C), whereas for *Porites*, this was at HI (28.1 ± 2.4°C; table 1). The breadth of the curve (*T*_br_) for *Acropora* was significantly larger at HI (9.2 ± 2.7°C) than at the other two locations (5.2 ± 0.4°C and 5.5 ± 2.1°C; ANOVA, *p* = 0.012; electronic supplementary material, table S4), consistent with the greater variability in temperature at HI. For the *Porites* populations, there was no significant variation in *T*_br_ among locations. The maximum Pn was highest at HI for both *Acropora* and *Porites* (table 1). Overall, the performance curves of the *Acropora* populations shifted vertically (through increased Pf_max_ at HI), horizontally (through increased *T*_opt_ at OI) and by changing the performance breadth (through increased *T*_br_ at HI). For the *Porites* populations, the performance curve shifted vertically (highest Pf_max_ at HI and lowest at OI) and horizontally (lowest *T*_opt_ at OI), but there was no change in the performance breadth.

**Table 1.** Average ± standard deviation of the parameter estimates for the physiological thermal response variables of *Acropora* spp*.* and *P. cylindrica* at HI, OI and LI computed through least square nonlinear regression for individual colonies (*n* = 5).

ther. resp.	parameter estimate	*Acropora* spp.			*Porites cylindrica*		
		HI	OI	LI	HI	OI	LI
Pn	*P*_max_ (O_2_ h^−1^ cm^−2^)	0.77 ± 0.16	0.30 ± 0.06	0.29 ± 0.07	1.16 ± 0.11	0.25 ± 0.02	0.64 ± 0.22
	*T*_opt_ (°C)	21.7 ± 2.0	27.8 ± 1.5	23.6 ± 3.9	28.1 ± 2.4	22.8 ± 3.3	26.0 ± 1.8
	*T*_br_ (°C)	9.2 ± 2.7	5.2 ± 0.4	5.5 ± 2.1	7.5 ± 2.4	9.2 ± 2.4	7.1 ± 1.4
*R*	*P*_max_ (O_2_ h^−1^ cm^−2^)	0.52 ± 0.03	0.44 ± 0.20	0.26 ± 0.08	0.78 ± 0.19	0.24 ± 0.12	0.38 ± 0.08
	*T*_opt_ (°C)	29.0 ± 0.5	39.6 ± 6.2	37.0 ± 10.3	30.5 ± 1.9	40.5 ± 22.8	28.6 ± 0.5
	*T*_br_ (°C)	8.2 ± 0.6	10.3 ± 4.4	11.5 ± 6.1	7.0 ± 1.5	14.4 ± 11.4	6.2 ± 0.7
*F*_v_/*F*_m_	*P*_max_ (no unit)	0.73 ± 0.01	0.72 ± 0.01	0.74 ± 0.03	0.69 ± 0.01	0.67 ± 0.03	0.70 ± 0.01
	*T*_opt_ (°C)	26.1 ± 0.4	25.9 ± 1.3	26.6 ± 0.7	26.0 ± 1.0	27.7 ± 0.9	25.5 ± 2.1
	*T*_br_ (°C)	16.7 ± 1.8	15.4 ± 4.2	10.7 ± 4.5	17.3 ± 1.4	15.2 ± 5.2	16.0 ± 6.5
rETR_m_	*P*_max_ (no unit)	123.3 ± 4.9	78.1 ± 7.1	52.1 ± 5.0	100.3 ± 5.7	83.6 ± 10.8	49.7 ± 8.3
	*T*_opt_ (°C)	23.7 ± 1.7	28.6 ± 0.4	29.4 ± 0.6	24.0 ± 2.7	29.2 ± 0.9	30.2 ± 0.6
	*T*_br_ (°C)	10.2 ± 1.7	3.6 ± 0.2	5.9 ± 1.1	9.4 ± 3.3	4.7 ± 0.7	6.8 ± 1.7

*R* rates of the *Acropora* (figure 2*d–f*) and *Porites* (figure 3*d*–*f*) populations at HI and LI increased with increasing temperature and then decreased at approximately *T*_exp_ + 3°C. At OI, *R* rates of the *Acropora* population increased linearly with temperature even at high temperatures, while the respiration rates of the *Porites* population were not strongly influenced by temperature. This resulted in relatively high *T*_opt_ estimates, ranging from 28.6 ± 0.5°C for the *Porites* population at HI up to 39.6 ± 6.2°C for the *Acropora* population at OI (table 1). We note that *T*_opt_ corresponds to the highest *R* rate which is generally interpreted to reflect metabolic costs (e.g. tissue maintenance, stress) rather than metabolic processes that contribute to growth. Caution must also be taken when interpreting the *R* rates, as declines in respiration at temperatures beyond *T*_opt_ are probably owing to impairment of the enzyme-driven reactions rather than a decrease in metabolic costs. *T*_br_ for respiration was relatively broad and not significantly different across locations for either species (electronic supplementary material, table S4). However, the *R* rate of the *Porites* population at HI was more than twofold higher compared with LI, and threefold higher compared with OI. Among the *Acropora* populations the variation in *P*_max_ was not significant (electronic supplementary material, table S4). Overall, the performance curve of the *Acropora* populations did not show any significant shift (either vertically or horizontally), while among *Porites* populations, the curve only shifted vertically (highest *T*_opt_ at HI).

### (d) Symbiont thermal response

Temperature did not have a strong effect on the *F*_v_/*F*_m_ of the *Acropora* populations (figure 2*g*–*i*) or *Porites* populations (figure 3*g*–*i*) at any of the study locations. This resulted in flattened performance curves, even though high experimental temperatures (*T*_exp_ + 4°C and *T*_exp_ + 5°C) caused a strong decline in *F*_v_/*F*_m_. Data points at 34°C of the *Acropora* population at LI were excluded when fitting the nonlinear regressions, because we did obtain reliable measurements. Nevertheless, the *T*_opt_ of both species was below the environmental summer temperature at every location. The variation in *T*_opt_ among the *Acropora* populations was not significantly different, ranging from 25.9 ± 1.3°C at OI to 26.6 ± 0.7°C at LI (table 1). There was slightly more variability in *T*_opt_ among the *Porites* populations, with a *T*_opt­_ at OI significantly higher than at LI (27.7 ± 0.9°C and 25.5 ± 2.1°C, respectively; Tukey post hoc, *p* = 0.038). *T*_br_ of both species were broad but became narrower with decreasing environmental variability, but this trend was not significant (electronic supplementary material, table S4). Lastly, *F*_v_/*F*_m_ was higher in *Acropora* than in *Porites*. Overall, the performance curve of *Acropora* did not change significantly among locations, while the performance curve of *Porites* only shifted horizontally (*T*_opt_ at OI the highest).

For the rETR_m_, *T*_opt_ significantly increased with environmental temperature for *Acropora* (figure 2*j*–*l*) and *Porites* (figure 3*j*–*l*). In addition, *T*_opt_ was also close to the environmental summer temperature for the populations at OI and LI, suggesting that acclimatization to the local temperature environment occurred at symbiont level for this particular photosynthesis trait. Likewise, *T*_br_ of both species were significantly larger at HI and smaller at OI and LI (table 1; electronic supplementary material, table S4), similar to the trend observed for *F*_v_/*F*_m_ and likely to be associated with the larger variability in environmental temperatures at HI. Lastly, rETR_m_ was highest at HI and lowest at LI (table 1). Overall, the performance curves of both species shifted vertically (highest Pf_max_ at HI), horizontally (lowest *T*_opt_ at HI) and in the performance breadth (widest *T*_br_ at HI).

### (e) Within-population variability

**Figure 4.**
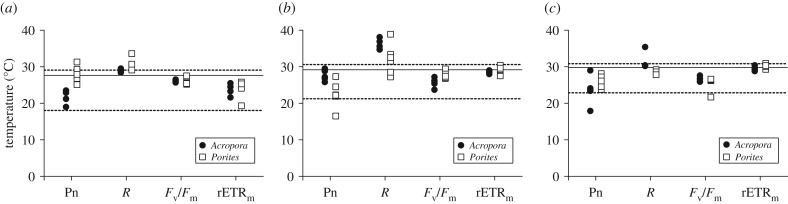
Variation in optimal temperature between colonies of *Acropora* spp. (closed circles) and *P. cylindrica* (open squares) at HI (*a*), OI (*b*) and LI (*c*) for net photosynthesis rate (Pn), respiration rate (*R*), photosynthetic efficiency (*F*_v_/*F*_m_) and maximum relative electron transport rate (rETR_m_). Datapoints are mean *T*_opt_ derived by nonlinear regression of four fragments from the same colony. Horizontal lines show the average seawater temperature measured over 14 days prior to the start of the thermal experiment, with dashed lines the minimum and maximum temperature of the previous year. Seawater temperature data sourced from AIMS 2017 [22].

There was strong model support for different thermal responses among locations, species and colonies (electronic supplementary material, table S3), indicating that the thermal performance varied considerably among colonies within species across all locations (electronic supplementary material, tables S5–S7). Regarding *T*_opt_ (figure 4), variability between colonies was generally larger for the holobiont responses compared to the symbiont responses. For instance, *T*_opt_ for Pn within the *Acropora* population at LI ranged from 17.9 to 29.0°C, while for *F*_v_/*F*_m_, this ranged only from 25.9 to 26.8°C within the same population. Similarly for *Porites*, the *T*_opt_ range for Pn within the population at HI was 6.2°C, while for *F*_v_/*F*_m_, this was only 2.3°C. Although these ranges are within the annual temperature range (electronic supplementary material, table S2), there were several *Porites* colonies with a *T*_opt_ above the maximum annual temperature (figure 4*a*). The variability in *T*_opt_ among *Porites* colonies was slightly larger than that observed for *Acropora*. Interestingly, the variability for Pn at OI was greatest among *Porites* colonies but smallest among *Acropora* colonies (respectively, 10.8 and 3.7°C; figure 4*b*) and vice versa at LI (figure 4*c*).

For most colonies of both species, *T*_opt_ values were within the range of the environmental variability (except for respiration, since that requires a different interpretation, as mentioned above). Generally, the *T*_opt_ of the holobiont performance were closer to the lower thermal threshold (only at HI was the *T*_opt_ of several *Porites* colonies above the upper threshold), while the *T*_opt­_ for the symbiont performances were closer to the average environmental temperature experienced during the weeks prior to the thermal experiment (solid line in figure 4*a*–*c*). These results suggest a higher capacity of acclimatization at symbiont compared with holobiont level, and poor performance of most colonies during summer in their local environments.

### (f) Chlorophyll concentration

**Figure 5.**
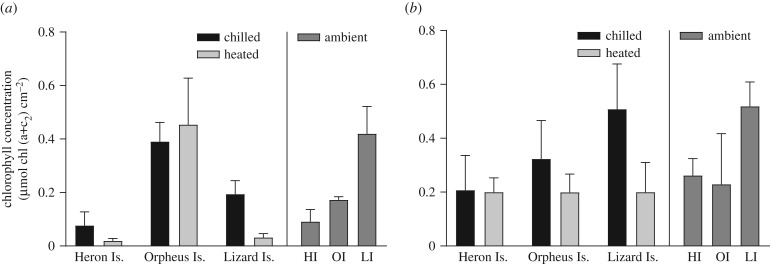
Average chlorophyll concentration in *Acropora* spp. (*a*) and *P. cylindrica* (*b*) after (chilled and heated, *n* = 10) and before (ambient, *n* = 5) exposure to a thermal gradient at HI, OI and LI. Error bars are standard deviation.

The thermal experiment affected the chlorophyll concentration in both species (mixed effect model with main effect of treatment for *Acropora* and *Porites*, respectively, *F*_1,38_ = 61.586, *p* < 0.001 and *F*_1,40_ = 24.950, *p* < 0.001), with generally a higher chlorophyll concentration in the fragments that were exposed to the chilled treatment than those exposed to the heated treatment (figure 5*a,b*). Only among the *Acropora* populations was there variation in the chlorophyll concentration between locations (mixed effect model with main effect of location for *Acropora*, *F*_2,12_ = 112.223, *p* < 0.001), with a higher concentration at OI possibly owing to the different *Acropora* species at this site (*A. valenciennesi* instead of *A. intermedia*). The chlorophyll concentration in fragments at ambient temperature was higher in *Porites* (figure 5*b*) than in *Acropora* (two-way ANOVA with main effect of species, *F*_1,27_ = 11.654, *p* = 0.002), which corresponds to the higher net photosynthetic performance observed with *Porites* fragments compared with *Acropora* fragments.

## 4. Discussion

Our study showed that the thermal performance varied between two coral species that occur across the same latitudinal gradient along the GBR, and that have broadly similar Indo-Pacific geographical distributions [49,50]. Moreover, our results indicate that both species are plastic thermal specialists, rather than thermal generalists, because the thermal performance differed within species among locations which could potentially be attributed to variation in symbiont types harboured within the coral populations at the different locations. Nevertheless, the observed differences in thermal performance among populations did not lead to an alignment of the optimal temperature for performance with the average temperature of the local environment. Therefore, we hypothesize that the capacity for thermal acclimatization of coral populations was constrained.

Thermal acclimatization of symbionts led to a closer alignment of the thermal performance with local environmental conditions compared to that which occurred at holobiont level. This was apparent by the performance curves fitted to the data segregated by location and species, as well as those fitted to the data segregated by colony. These results are consistent with Howells *et al*. [51] who showed that *Symbiodinium* species from warm environments maintained greater photosynthetic performance at high temperatures than the same species from cooler environments. However, as we did not identify the *Symbiodinium* types in our study, it is also possible that different symbiont species with different physiological traits were present among the coral populations (e.g. [52]). For instance, the *Acropora* corals at HI may harbour a different symbiont population than the corals at lower (and warmer) latitudes [53]. Further research is required to determine whether acclimatization at the symbiont level observed here was owing to differences in *Symbiodinium* types, or owing to local acclimatization and/or adaptation of local populations of the same symbiont type.

Regarding the symbiont traits more specifically, *T*_opt_ and *T*_br_ for rETR_m_ increased with average environmental temperature and variability*,* according to our hypotheses. For *F*_v_/*F*_m_, the *T*_br_ increased with environmental thermal variability, while the *T*_opt_ were below the average environmental temperatures and remarkably similar between locations for both coral species. These different results for different symbiont traits suggest that the effect of temperature on photosynthesis is sequential instead of simultaneous: where the electron transport rate is reduced at increased temperature, this could prevent inhibition of the maximum quantum yield. This interpretation is based on other studies which showed that during the early stages of thermal stress, the enzyme activity in the Calvin–Benson cycle is slower, which directly influences the rate of electron transport but does not directly damage the photosystems (see review by Allakhverdiev *et al*. [54]). Additional research is required to assess functional differences between coral symbiont species, and whether and how different symbiont species make variable contributions to coral host energetics.

We hypothesized that the TPCs of subpopulations of plastic thermal specialist species would change shape and position according to the thermal variability and mean environmental temperature of their local environment, as observed for various physiological traits of other coral species (e.g. [55,56]). Specifically, we expected increasing thermal breadth with increasing latitude owing to greater thermal heterogeneity at high latitudes, but decreasing thermal optima with increasing latitude owing to lower mean environmental temperatures. Results for the thermal performance of symbiont traits showed a general trend consistent with these hypotheses, but not for the thermal performance of holobiont traits. In fact, although the optimal temperature for holobiont performance (Pn and *R*) varied among coral populations, it did not consistently match the (recent) average environmental temperatures at each site as previously observed for the temperate coral *Oculina patagonica* [29]. Instead, *T*_opt_ was below the environmental temperature at all three locations (*R* excluded), except for the *Porites* population at HI. Thermal acclimatization along a latitudinal cline of the photosynthetic performance specifically has been observed for a variety of organisms. For instance, a positive correlation between latitude and *T*_opt_ for photosynthesis has been observed for macrophytes [57]. Similarly, *T*_opt_ for net photosynthesis was higher in tropical tree species than in temperate tree species [58]. However, the absence of a correlation between latitude and *T*_opt_ for photosynthesis for corals has now been reported in three studies [29,59]. Collectively, these findings suggest the presence of factors that constrain thermal acclimatization of local coral populations more so than for other taxa.

Despite the observed mismatch between *T*_opt_ and local mean environmental temperatures, the *T*_br_ of each population was wide and generally encompassed the range of temperatures experienced at each location. This means that corals live at suboptimal conditions for performance, but declines in performance at temperatures above and below the optima are relatively small. Similarly, wide performance breadths are observed previously on corals [29,60,61], suggesting that this finding is not species-specific. Broad *T*_br_ could explain why *T*_opt_ did not consistently match the average environmental temperatures because the small increase in performance achieved through ‘perfect’ acclimatization of the thermal response might not outweigh the costs of acclimatization. However, the observed *T*_br_ (presented as the average across multiple coral colonies at each location) also reflects the high level of variation in performance among colonies. A likely explanation for this high among-colony variation is dispersal of coral larvae across large distances, and among subpopulations with different thermal histories. Coral recruits can be sourced from the local reef [62], but many spawning species (including the species studied here) produce larvae with a relatively long planktonic stage that can disperse to maintain moderate to high levels of gene flow along the GBR [30]. Hence, the influx of maladapted (cold or warm) genotypes or phenotypes on reefs around LI, OI and HI may have prevented perfect acclimatization of each population and increased within-population variation. Moreover, despite collection of coral fragments from colonies that were approximately the same size, these colonies potentially settled onto the reef in different years with different environmental conditions. Strong developmental plasticity at the time of settlement could also drive high variation in thermal responses later observed among adult colonies. Lastly, the variation in *T*_opt_ for holobiont dominated responses between *Acropora* colonies was larger than the thermal variation they experience annually. Although this negates successful acclimatization of the overall population performance, the silver lining is that this high level of natural variation in thermal performance provides raw material for natural selection and adaptation and can therefore promote survival under climate change. In addition, the notion that the performance curves at symbiont level appear better acclimated to the local environment supports the idea that maladapted immigrated colonies are able to take up well-acclimated/adapted symbionts from the local environment.

The respiration, or oxygen consumption, rate represents the whole-organism metabolism, although symbiont respiration is considered to be negligible, as the symbiont : coral ratio generally ranges somewhere between 0.03 and 0.1 depending on the coral species [35,63]. Thus, the observed changes in the respiration rate in this study were mostly owing to changes in the host physiology. For corals, interpretation of the thermal optima for respiration rate is complicated. For instance, *T*_opt_ were well above 30°C for most populations considered here, temperatures only rarely experienced in the environment. Within the temperature range that corals can tolerate, respiration is often found to increase with increasing temperature [64]. Therefore, it is likely that the performance curve for respiration is asymmetrical, with a sharp sudden decrease in the respiration rate close to the upper thermal threshold. Indeed, a recent study demonstrated such a left-skewed performance curve for the respiration rates of the temperate coral *Astrangia poculata* [55]. Additionally, high respiration rates are generally associated with high levels of stress and metabolic costs [65], suggesting that the parameter estimation for *T*_opt_ signified the temperature at which metabolic costs were highest rather than the temperature at which performance was maximized. We observed declined respiration rates for the populations at HI after approximately 30°C, but at OI and LI, respiration declined only at the highest two temperatures measured (greater than 33°C). This suggests that the latitudinal thermal cline influenced the thermal acclimatization to some extent. Further research encompassing a wider temperature scope, and during which cellular responses are monitored in addition to whole-organism respiration rates will provide more insight into the true shape of the curve.

In summary, our findings show that the holobiont thermal performance varied among locations and between species, therefore excluding a thermal generalist strategy, although thermal specialization through acclimated *T*_opt_ and narrow *T*_br_ was neither observed. Instead, populations of both species across all locations generally lived at temperatures above their optima, constraining their performance nearly all year round. While these temperatures may not be lethal to the corals in the short term, they are suboptimal for fitness which may significantly reduce their resilience to future summer extremes.

## Supplementary Material

Supplementary data - Tables S1 - S7

## Supplementary Material

Supplementary figure - Figure S1

## Data accessibility

The raw data is accessible at the Tropical Data Hub: http://dx.doi.org/10.25903/5cbfd123d7a91.
