# Supplementary material for: Thermal performance of scleractinian corals along a latitudinal gradient on the Great Barrier Reef
Source: Philos Trans R Soc Lond B Biol Sci. 2019 Jun 17;374(1778):20180546. doi: 10.1098/rstb.2018.0546 (PMC6606464; doi:10.1098/rstb.2018.0546)
Supplement: Supplementary data - Tables S1 - S7 [file rstb20180546supp1.docx]

**Table S1** Mean (and maximum) monthly photosynthetically active irradiance (µmol photons s^-1^ m^-2^) for Jan 2016 to March 2017 at Heron Island, Orpheus Island and Lizard Island measured at the surface. Davies Reef is situated at a similar latitude as Orpheus Island (18° S) and included as an indication of the irradiance attenuation at 1.9 m depth. Shaded areas highlight the irradiance prior to and during the thermal experiment at the specific sites. Data sourced from the Australian Institute of Marine Science data portal ([www.aims.gov.au/docs/data/data.html](http://www.aims.gov.au/docs/data/data.html)).

|  | Heron Island  (surface) | Orpheus Island  (surface) | Lizard Island  (surface) | Davies Reef  (1.9 m) |
| --- | --- | --- | --- | --- |
| Jan 2016 | 602 (2351) | 489 (1973) | 539 (2083) | 224 (988) |
| Feb | 510 (2287) | 459 (1874) | 520 (2060) | 206 (979) |
| Mar | 468 (2116) | 344 (2096) | 431 (2070) | 138 (881) |
| Apr | 413 (1904) | 351 (1722) | 393 (1933) | 118 (741) |
| May | 370 (1706) | 275 (1581) | 336 (1633) | 88 (770) |
| Jun | 299 (1445) | 247 (1541) | 321 (1552) | 43 (587) |
| Jul | 325 (1577) | 267 (1329) | 312 (1594) | 37 (542) |
| Aug | 371 (1824) | 316 (1591) | 369 (1654) | 71 (742) |
| Sep | 447 (1887) | 368 (1619) | 447 (1829) | 104 (802) |
| Oct | 522 (1982) | 430 (1770) | 501 (1897) | 61 (787) |
| Nov | 566 (2154) | 459 (1718) | 517 (1934) | 100 (876) |
| Dec | 571 (2182) | 440 (1841) | 502 (2066) | 98 (802) |
| Jan 2017 | 532 (2267) | 387 (1858) | 428 (1969) | 95 (681) |
| Feb | 558 (2144) | 394 (2148) | 388 (1949) | 114 (752) |
| Mar | 405 (2041) | 352 (1843) | 409 (1908) | 88 (688) |

**Table S2** Summary of the seawater temperatures at Heron Island (measured at 5.4 m depth), Orpheus Island (measured at 5.8 m depth) and Lizard Island (measured at 10.1 m depth) analysed over the period December 1 to March 31 in the years 2015 – 2016 (first three columns) and 2016 – 2017 (last three columns). Underscore shows the data that defined the thermal environment prior to the thermal experiment at each location. At Lizard Island in 2015 – 2016, data was not recorded during December and January. Data sourced from the Australian Institute of Marine Science data portal ([www.aims.gov.au/docs/data/data.html](http://www.aims.gov.au/docs/data/data.html)).

|  |  |  | **2015-2016** |  |  | **2016-2017** |  |
| --- | --- | --- | --- | --- | --- | --- | --- |
|  |  | HI | OI | LI | HI | OI | LI |
| Mean T ± sd (°C) | Dec | 25.6 ± 0.4 | 28.7 ± 0.2 | n.a | 26.0 ± 0.3 | 28.7 ± 0.2 | 28.4 ± 0.1 |
|  | Jan | 26.6 ± 0.3 | 29.4 ± 0.2 | n.a | 26.8 ± 0.3 | 29.8 ± 0.2 | 29.5 ± 0.1 |
|  | Feb | 27.2 ± 0.3 | 29.3 ± 0.3 | 29.8 ± 0.1 | 27.6 ± 0.3 | 29.8 ± 0.1 | 29.7 ± 0.1 |
|  | Mar | 27.2 ± 0.4 | 29.2 ± 0.1 | 30.1 ± 0.1 | 27.6 ± 0.3 | 29.8 ± 0.2 | 29.9 ± 0.1 |
| Min – Max T (°C) | Dec | 22.8 - 27.1 | 27.3 - 29.6 | n.a | 24.0 - 27.7 | 26.5 - 29.7 | 27.8 - 29.2 |
|  | Jan | 24.2 - 28.8 | 28.4 - 30.4 | n.a | 25.2 - 28.5 | 29.0 - 31.0 | 28.6 - 30.5 |
|  | Feb | 25.5 - 28.8 | 26.7 - 30.6 | 29.3 - 30.7 | 25.7 - 29.1 | 29.4 - 30.8 | 29.0 - 30.6 |
|  | Mar | 25.6 - 28.5 | 28.3 - 30.1 | 29.5 - 30.8 | 26.3 - 28.9 | 29.2 - 30.9 | 29.2 - 30.8 |
| Annual T range (°C) | | 10.5 | 9.4 | 8.0 | 11.0 | 8.7 | 6.5 |
| % hrs > 28 ⁰C | Dec | 0 | 97 | n.a | 0 | 99 | 94 |
|  | Jan | 2 | 100 | n.a | 0 | 100 | 100 |
|  | Feb | 2 | 93 | 100 | 13 | 100 | 100 |
|  | Mar | 1 | 100 | 100 | 15 | 100 | 100 |
| % hrs > 29 ⁰C | Dec | 0 | 29 | n.a | 0 | 24 | 1 |
|  | Jan | 0 | 83 | n.a | 0 | 100 | 82 |
|  | Feb | 0 | 77 | 100 | 0 | 100 | 100 |
|  | Mar | 0 | 65 | 100 | 0 | 100 | 100 |
| % hrs > 30 ⁰C | Dec | 0 | 0 | n.a | 0 | 0 | 0 |
|  | Jan | 0 | 4 | n.a | 0 | 30 | 12 |
|  | Feb | 0 | 6 | 24 | 0 | 13 | 13 |
|  | Mar | 0 | 0 | 68 | 0 | 21 | 39 |

**Table S3** Comparison of thermal performance curves with different combinations of data selection for different physiological responses. Nonlinear regression models were fitted to the data as follows: 1) location, species and colony variability pooled together, referred to as “all data”; 2) only variability by location; 3) only variability by species; 4) variability by species and location; 5) species, location and within-population variability. K is number of estimated parameters in the model, delta AIC is the difference between the AIC value of the model and the minimum AIC value among all the models of the thermal response and the AIC weight is the weighted average of the model and represent the relative likelihood.

| Thermal response | Data selection | K | Cumulative AIC | Δ AIC |
| --- | --- | --- | --- | --- |
| Pnet | All data | 3 | 657.33 | 1678.90 |
|  | Location | 9 | -268.93 | 752.64 |
|  | Species | 6 | 461.25 | 1482.82 |
|  | Location * Species | 18 | -477.24 | 544.33 |
|  | Location * Species * Colony | 93 | -1021.57 | 0.00 |
| R | All data | 3 | -241.40 | 1649.48 |
|  | Location | 9 | -1304.05 | 586.83 |
|  | Species | 6 | -360.57 | 1530.31 |
|  | Location * Species | 18 | -1764.21 | 126.68 |
|  | Location * Species * Colony | 90 | -1890.88 | 0.00 |
| F_v_/F_m_ | All data | 3 | -2654.58 | 432.15 |
|  | Location | 9 | -2698.01 | 388.72 |
|  | Species | 6 | -2803.05 | 283.68 |
|  | Location * Species | 18 | -2879.31 | 207.42 |
|  | Location * Species * Colony | 93 | -3086.73 | 0.00 |
| rETR_m_ | All data | 3 | 7328.03 | 2487.53 |
|  | Location | 9 | 6490.26 | 1649.75 |
|  | Species | 6 | 7298.87 | 2458.37 |
|  | Location * Species | 18 | 6397.89 | 1557.38 |
|  | Location * Species * Colony | 93 | 4840.51 | 0.00 |

**Table S4** Results of the statistical analyses to detect population variability (at Lizard Island, Orpheus Island and Heron Island) in the parameter estimates (P_max_, T_opt_ and T_br_) of the thermal performance curves for four physiological response variables. Parameter estimates were calculated using non-linear regression for 5 colonies at each location for *Acropora* spp. and *Porites cylindrica*.

| Thermal response | Parameter estimate | df | *Acropora* F-value | *p-value* | df | *Porites*  F-value | *p-value* |
| --- | --- | --- | --- | --- | --- | --- | --- |
| Pnet | P_max_ | 2, 12 | 35.26 | 0.000 | 2, 14 | 68.61 | 0.000 |
|  | T_opt_ | 2, 12 | 7.21 | 0.009 | 2, 14 | 5.87 | 0.014 |
|  | T_br_ | 2, 12 | 6.51 | 0.012 | 2, 14 | 1.69 | 0.220 |
| R | P_max_ | 2, 11 | 3.70 | 0.059 | 2, 14 | 23.99 | 0.000 |
|  | T_opt_ | 2, 11 | 3.00 | 0.091 | 2, 14 | 1.12 | 0.354 |
|  | T_br_ | 2, 11 | 0.59 | 0.572 | 2, 14 | 2.20 | 0.148 |
| F_v_/F_m_ | P_max_ | 2, 12 | 1.27 | 0.315 | 2, 14 | 1.55 | 0.247 |
|  | T_opt_ | 2, 12 | 0.71 | 0.511 | 2, 14 | 4.32 | 0.034 |
|  | T_br_ | 2, 12 | 3.21 | 0.077 | 2, 14 | 0.28 | 0.763 |
| rETR_m_ | P_max_ | 2, 12 | 160.83 | 0.000 | 2, 14 | 42.48 | 0.000 |
|  | T_opt_ | 2, 12 | 45.96 | 0.000 | 2, 14 | 22.27 | 0.000 |
|  | T_br_ | 2, 12 | 46.12 | 0.000 | 2, 14 | 8.13 | 0.004 |

**Table S5** Parameter estimates (P_max_, T_opt_ and T_br_) for the individual *Acropora* and *Porites* colonies around **Heron Island** for four physiological response variables (net photosynthesis rate, respiration rate, photosynthetic efficiency and electron transport rate). Non-linear regressions were fitted to the data of 4 fragments from the same colony. *Acropora* colony A.51 was excluded at the start of the experiment due to paleness and replaced by the same amount of extra fragments of colony A.52.

| Thermal | Param. | **Heron Island *Acropora* population** | | | | | **Heron Island *Porites* population** | | | | |
| --- | --- | --- | --- | --- | --- | --- | --- | --- | --- | --- | --- |
| response | estim. | A.51 | A.52 | A.53 | A.54 | A.55 | P.51 | P.52 | P.53 | P.54 | P.55 |
| Pnet | P_max_ | Excluded | 0.73 ± 0.08 | 0.61 ± 0.26 | 0.75 ± 0.09 | 0.99 ± 0.19 | 1.02 ± 0.11 | 1.25 ± 0.14 | 1.27 ± 0.13 | 1.18 ± 0.11 | 1.06 ± 0.08 |
|  | T_opt_ | Excluded | 23.5 ± 3.4 | 19.0 ± 19.7 | 22.9 ± 4.8 | 21.2 ± 4.7 | 29.3 ± 1.7 | 26.8 ± 1.5 | 27.9 ± 0.6 | 31.3 ± 4.1 | 25.1 ± 3.4 |
|  | T_br_ | Excluded | 7.5 ± 3.5 | 13.1 ± 15.7 | 8.7 ± 5.0 | 7.4 ± 3.3 | 6.6 ± 2.6 | 7.4 ± 3.3 | 4.2 ± 0.8 | 9.2 ± 5.1 | 10.3 ± 6.5 |
| R | P_max_ | Excluded | 0.54 ± 0.02 | 0.48 ± 0.02 | 0.55 ± 0.03 | 0.51 ± 0.02 | 0.59 ± 0.03 | 1.09 ± 0.20 | 0.70 ±0.04 | 0.81 ± 0.05 | 0.73 ± 0.03 |
|  | T_opt_ | Excluded | 29.1 ± 0.5 | 28.8 ± 0.9 | 29.5 ± 1.1 | 28.4 ± 0.6 | 29.6 ± 0.8 | 33.6 ± 5.6 | 29.3 ± 0.6 | 30.7 ± 1.2 | 29.1 ± 1.1 |
|  | T_br_ | Excluded | 7.9 ± 1.0 | 9.1 ± 2.1 | 7.7 ± 1.8 | 8.1 ± 1.3 | 6.7 ± 1.2 | 8.4 ± 4.3 | 5.0 ± 0.8 | 6.3 ± 1.3 | 8.7 ± 2.2 |
| F_v_/F_m_ | P_max_ | Excluded | 0.72 ± 0.01 | 0.74 ± 0.01 | 0.73 ± 0.01 | 0.74 ± 0.01 | 0.68 ± 0.01 | 0.67 ± 0.01 | 0.69 ± 0.01 | 0.69 ± 0.01 | 0.70 ± 0.01 |
|  | T_opt_ | Excluded | 25.9 ± 0.7 | 25.7 ± 1.0 | 26.2 ± 0.7 | 26.5 ± 0.8 | 26.5 ± 1.4 | 27.5 ± 1.0 | 25.2 ± 1.7 | 25.6 ± 0.8 | 25.4 ± 1.3 |
|  | T_br_ | Excluded | 17.4 ± 2.7 | 18.9 ± 3.8 | 15.1 ± 2.8 | 15.4 ± 3.2 | 18.9 ± 6.8 | 17.8 ± 5.3 | 18.0 ± 5.5 | 15.4 ± 2.6 | 16.4 ± 4.2 |
| rETR_m_ | P_max_ | Excluded | 120.0 ± 6.3 | 119.1 ± 10.6 | 124.4 ± 6.1 | 129.7 ± 9.7 | 96.7 ± 4.9 | 105.3 ± 4.8 | 92.5 ± 6.4 | 101.1 ± 6.1 | 105.9 ± 14.8 |
|  | T_opt_ | Excluded | 24.5 ± 2.0 | 21.6 ± 5.9 | 23.3 ± 2.8 | 25.5 ± 2.2 | 25.6 ± 0.7 | 25.8 ± 1.2 | 24.1 ± 4.2 | 25.3 ± 1.1 | 19.3 ± 8.1 |
|  | T_br_ | Excluded | 8.9 ± 2.9 | 12.5 ± 6.5 | 10.4 ± 3.3 | 9.0 ± 4.2 | 6.3 ± 1.1 | 8.9 ± 2.4 | 10.9 ± 6.6 | 6.8 ± 1.6 | 14.3 ± 7.3 |

**Table S6** Parameter estimates (P_max_, T_opt_ and T_br_) for the individual *Acropora* and *Porites* colonies around **Orpheus Island** for four physiological response variables (net photosynthesis rate, respiration rate, photosynthetic efficiency and electron transport rate). Non-linear regressions were fitted to the data of 4 fragments from the same colony (for colonies A.11 & A.18 of the *Acropora* population*,* and P.12 &P.16 of the *Porites* population, regressions were fitted to only 2 fragments of the same colony).

| Thermal | Param. | **Orpheus Island *Acropora* population** | | | | | | **Orpheus Island *Porites* population** | | | | | |
| --- | --- | --- | --- | --- | --- | --- | --- | --- | --- | --- | --- | --- | --- |
| response | estim. | A.5 | A.6 | A.8 | A.11 | A.15 | A.18 | P.12 | P.16 | P.20 | P.31 | P.32 | P.33 |
| Pnet | P_max_ | 0.27 ± 0.02 | 0.20 ± 0.01 | 0.34 ± 0.03 | 0.36 ± 0.04 | 0.32 ± 0.03 | 0.32 ± 0.03 | 0.26 ± 0.02 | 0.21 ± 0.01 | 0.23 ± 0.03 | 0.26 ± 0.01 | 0.24 ± 0.03 | 0.28 ± 0.10 |
|  | T_opt_ | 25.8 ± 1.1 | 26.8 ± 0.6 | 29.0 ± 0.6 | 29.5 ± 1.0 | 27.2 ± 0.8 | 28.8 ± 0.9 | 24.5 ± 1.2 | 27.3 ± 0.8 | 22.3 ± 6.8 | 24.5 ± 1.0 | 21.9 ± 3.9 | 16.5 ± 8.7 |
|  | T_br_ | 11.2 ± 2.6 | 11.0 ± 1.8 | 9.0 ± 1.8 | 10.4 ± 3.0 | 10.4 ± 2.2 | 11.0 ± 3.0 | 10.8 ± 2.0 | 21.2 ± 4.4 | 24.4 ± 13.8 | 14.2 ± 2.0 | 18.4 ± 6.0 | 22.4 ± 8.8 |
| R | P_max_ | 0.28 ± 0.09 | 0.84 ± 0.21 | 0.38 ± 0.07 | 0.37 ± 0.07 | 0.44 ± 1.12 | 0.36 ± 0.25 | 0.15 ± 0.01 | 0.24 ± 0.08 | 0.17 ± 0.01 | 0.20 ± 0.01 | 0.23 ± 0.01 | 0.21 ± 0.02 |
|  | T_opt_ | 38.1 ± 8.3 | 36.9 ± 7.0 | 35.6 ± 3.2 | 34.7 ± 3.8 | 51.5 ± 78.7 | 40.6 ± 14.5 | 27.2 ± 1.5 | 38.9 ± 10.0 | 33.4 ± 3.1 | 28.5 ± 0.7 | 31.5 ± 5.0 | 32.5 ± 5.2 |
|  | T_br_ | 20.6 ± 9.6 | 11.4 ± 8.8 | 13.0 ± 3.8 | 12.4 ± 4.8 | 36.8 ± 64.8 | 20.6 ± 13.4 | 17.0 ± 6.6 | 23.2 ± 11.8 | 18.2 ± 6.2 | 15.0 ± 3.4 | 25.6 ± 19.6 | 22.6 ± 14.8 |
| F_v_/F_m_ | P_max_ | 0.72 ± 0.01 | 0.71 ± 0.01 | 0.73 ± 0.01 | 0.72 ± 0.01 | 0.72 ± 0.01 | 0.74 ± 0.01 | 0.66 ± 0.01 | 0.63 ± 0.02 | 0.66 ± 0.01 | 0.70 ± 0.01 | 0.71 ± 0.01 | 0.64 ± 0.02 |
|  | T_opt_ | 26.2 ± 0.7 | 25.4 ± 0.7 | 27.2 ± 0.6 | 23.7 ± 4.0 | 26.0 ± 1.3 | 27.2 ± 0.7 | 27.8 ± 0.6 | 29.4 ± 2.0 | 28.0 ± 0.3 | 26.8 ± 1.3 | 27.3 ± 1.1 | 27.4 ± 0.7 |
|  | T_br_ | 20.0 ± 2.6 | 25.2 ± 2.6 | 30.0 ± 4.6 | 43.2 ± 17.6 | 57.6 ± 8.4 | 29.2 ± 5.4 | 25.8 ± 4.6 | 33.2 ± 18.0 | 17.0 ± 1.6 | 40.6 ± 11.6 | 45.6 ± 12.6 | 19.2 ± 3.6 |
| rETR_m_ | P_max_ | 84.0 ± 4.4 | 88.2 ± 3.6 | 76.8 ± 4.2 | 68.0 ± 5.2 | 76.6 ± 4.4 | 74.7 ± 6.2 | 106 ± 9.0 | 73.1 ± 4.7 | 82.2 ± 8.4 | 79.1 ± 5.2 | 84.9 ± 6.2 | 76.4 ± 3.8 |
|  | T_opt_ | 28.4 ± 0.3 | 29.0 ± 0.2 | 28.6 ± 0.3 | 28.7 ± 0.3 | 28.7 ± 0.3 | 28.0 ± 0.4 | 27.5 ± 0.5 | 30.4 ± 0.6 | 29.4 ± 0.9 | 29.1 ± 0.5 | 29.0 ± 0.6 | 29.6 ± 0.3 |
|  | T_br_ | 8.0 ± 0.8 | 7.2 ± 0.4 | 7.2 ± 0.6 | 6.6 ± 0.8 | 7.2 ± 0.6 | 7.6 ± 1.0 | 8.8 ± 1.4 | 9.8 ± 1.4 | 11.0 ± 2.8 | 9.6 ± 1.4 | 11.0 ± 2.0 | 7.4 ± 0.6 |

**Table S7** Parameter estimates (P_max_, T_opt_ and T_br_) for the individual *Acropora* and *Porites* colonies around **Lizard Island** for four physiological response variables (net photosynthesis rate, respiration rate, photosynthetic efficiency and electron transport rate). Non-linear regressions were fitted to the data of 4 fragments from the same colony. There are no parameter estimates for the respiration rate of *Acropora* colony A.43, because the Gaussian distribution did not fit the data.

| Thermal | Param. | **Lizard Island *Acropora* population** | | | | | **Lizard Island *Porites* population** | | | | |
| --- | --- | --- | --- | --- | --- | --- | --- | --- | --- | --- | --- |
| response | estm. | A.41 | A.42 | A.43 | A.44 | A.45 | P.41 | P.42 | P.43 | P.44 | P.45 |
| Pnet | P_max_ | 0.26 ± 0.02 | 0.34 ± 0.16 | 0.19 ± 0.03 | 0.26 ± 0.03 | 0.37 ± 0.04 | 0.31 ± 0.02 | 0.56 ± 0.03 | 0.69 ± 0.06 | 0.80 ± 0.06 | 0.86 ± 0.05 |
|  | T_opt_ | 23.4 ± 1.4 | 17.9 ± 8.7 | 29.0 ± 0.6 | 23.7 ± 2.5 | 24.1 ± 1.4 | 23.8 ± 3.3 | 28.1 ± 0.5 | 27.2 ± 1.0 | 24.6 ± 2.1 | 26.1 ± 0.7 |
|  | T_br_ | 5.3 ± 1.2 | 8.4 ± 4.3 | 2.6 ± 0.9 | 5.9 ± 2.3 | 5.2 ± 1.3 | 9.0 ± 3.8 | 6.7 ± 1.0 | 5.9 ± 1.6 | 8.1 ± 2.7 | 5.9 ± 1.0 |
| R | P_max_ | 0.20 ± 0.01 | 0.24 ± 0.01 | n.a. | 0.22 ± 0.09 | 0.37 ± 0.79 | 0.26 ± 0.02 | 0.37 ± 0.02 | 0.39 ± 0.03 | 0.44 ± 0.03 | 0.46 ± 0.03 |
|  | T_opt_ | 30.2 ± 0.7 | 30.4 ± 2.2 | n.a. | 35.4 ± 11.6 | 52.1 ± 77.3 | 28.7 ± 0.9 | 28.6 ± 0.5 | 29.2 ± 0.9 | 28.6 ± 0.5 | 27.8 ± 0.5 |
|  | T_br_ | 6.6 ± 0.9 | 9.2 ± 3.3 | n.a. | 9.9 ± 7.8 | 20.4 ± 33.6 | 7.5 ± 1.9 | 5.9 ± 0.8 | 6.3 ± 1.4 | 5.8 ± 0.8 | 5.7 ± 0.8 |
| F_v_/F_m_ | P_max_ | 0.78 ± 0.02 | 0.72 ± 0.01 | 0.72 ± 0.01 | 0.72 ± 0.00 | 0.77 ± 0.02 | 0.70 ± 0.01 | 0.68 ± 0.01 | 0.70 ± 0.01 | 0.70 ± 0.01 | 0.69 ± 0.01 |
|  | T_opt_ | 26.8 ± 0.5 | 25.9 ± 0.6 | 27.6 ± 1.9 | 26.0 ± 0.8 | 26.7 ± 0.5 | 26.2 ± 0.7 | 26.3 ± 1.0 | 26.5 ± 0.6 | 21.7 ± 5.0 | 26.6 ± 0.5 |
|  | T_br_ | 7.1 ± 0.9 | 10.3 ± 1.4 | 11.2 ± 6.2 | 17.9 ± 3.8 | 7.0 ± 0.9 | 13.4 ± 1.9 | 15.7 ± 3.0 | 10.1 ± 1.2 | 27.1 ± 10.0 | 13.8 ± 1.5 |
| rETR_m_ | P_max_ | 46.4 ± 2.5 | 51.9 ± 3.7 | 48.3 ± 3.0 | 58.7 ± 5.8 | 55.2 ± 3.6 | 35.3 ± 2.3 | 56.6 ± 3.9 | 51.4 ± 3.3 | 51.9 ± 1.9 | 53.7 ± 3.8 |
|  | T_opt_ | 28.9 ± 0.8 | 28.8 ± 0.9 | 30.4 ± 0.8 | 29.6 ± 1.6 | 29.1 ± 0.9 | 29.3 ± 1.4 | 30.0 ± 1.0 | 30.6 ± 0.7 | 30.9 ± 1.0 | 30.3 ± 0.8 |
|  | T_br_ | 7.0 ± 1.7 | 6.4 ± 1.7 | 4.1 ± 1.3 | 6.0 ± 2.3 | 6.0 ± 1.5 | 9.3 ± 3.8 | 6.6 ± 1.6 | 5.2 ± 0.9 | 7.8 ± 1.4 | 5.4 ± 1.0 |
