# Supplementary figures and images for: Thermal performance of scleractinian corals along a latitudinal gradient on the Great Barrier Reef
Source: Philos Trans R Soc Lond B Biol Sci. 2019 Jun 17;374(1778):20180546. doi: 10.1098/rstb.2018.0546 (PMC6606464; doi:10.1098/rstb.2018.0546)

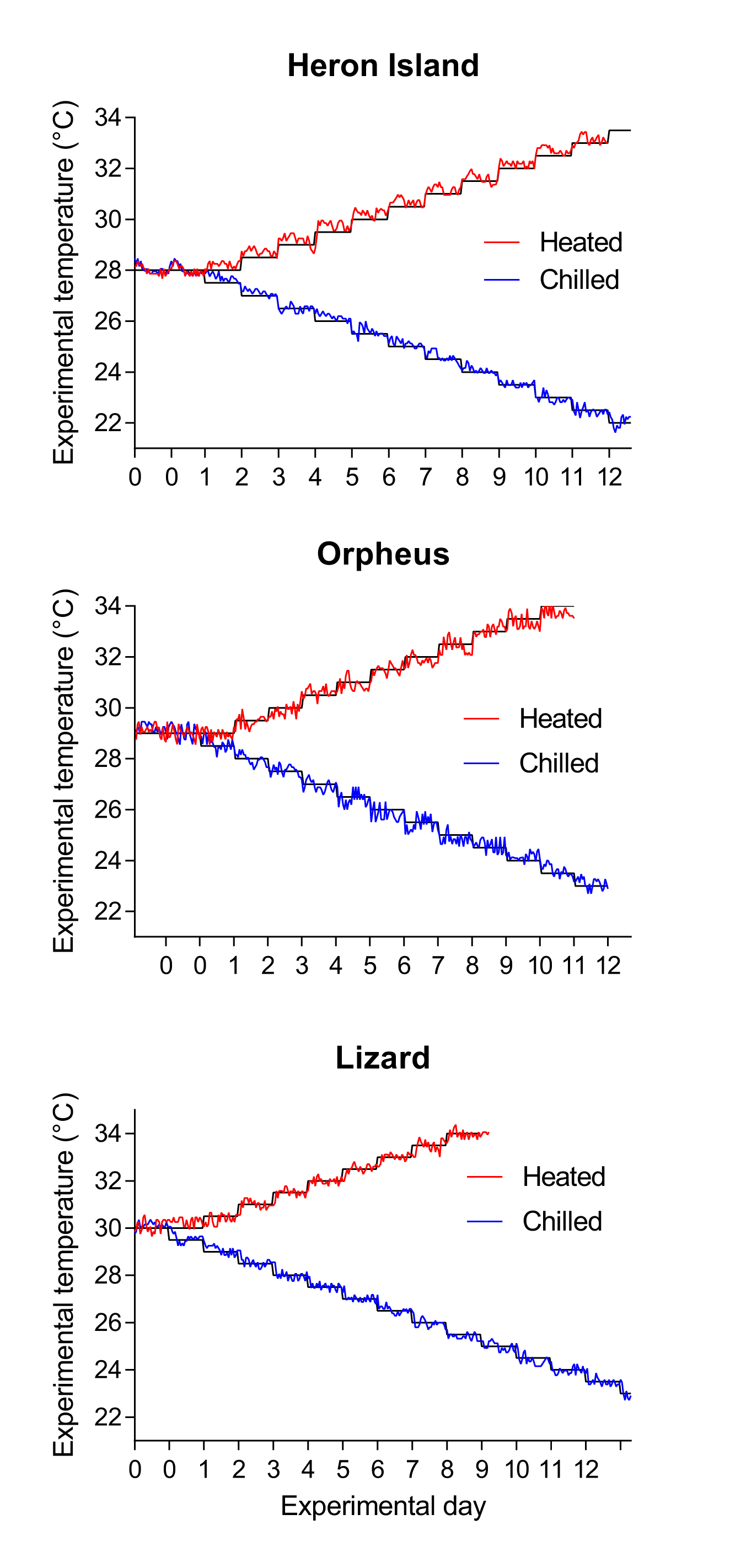

Supplement: Supplementary figure - Figure S1 [file rstb20180546supp2.tif]
